# A blood-biomarker based CTC-ALRI score predicts recurrence in hepatocellular carcinoma patients following curative resection

**DOI:** 10.3389/fonc.2026.1788776

**Published:** 2026-03-16

**Authors:** Jinfeng Wang, Guangjie Han, Xin Han, Jianfei Shi, Lili Mi, Ning Li, Man Zhao, Xiaoling Duan, Fei Yin

**Affiliations:** Department of Gastroenterology, the Fourth Hospital of Hebei Medical University, Shijiazhuang, Hebei, China

**Keywords:** aspartate aminotransferase-to-lymphocyte ratio index, circulating tumor cell, curative resection, hepatocellular carcinoma, postoperative recurrence

## Abstract

**Background:**

Hepatocellular carcinoma (HCC) recurrence after curative resection remains a critical challenge that significantly compromises long-term survival. This study aimed to develop a novel blood-based prognostic scoring system integrating circulating tumor cells (CTCs) and aspartate aminotransferase-to-lymphocyte ratio index (ALRI) for predicting postoperative HCC recurrence.

**Methods:**

This retrospective study enrolled 160 HCC patients undergoing curative resection between January 2019 and July 2023. CTCs were detected using the Cyttel method with immunomagnetic bead negative enrichment combined with immunofluorescence *in situ* hybridization. COX regression identified independent factors for recurrence-free survival (RFS). Patients were stratified into three groups (CTC-ALRI scores 0, 1, 2) based on preoperative CTC status and ALRI levels. Prognostic performance was evaluated using Kaplan-Meier analysis, Cox regression, logistic regression, receiver operating characteristic curves and time-independent C-index.

**Results:**

Multivariate Cox regression identified baseline ALRI and CTC as independent predictors of RFS and were used to develop the CTC-ALRI score. Recurrence rates increased progressively across score groups: 24.00% (score 0), 52.11% (score 1), and 84.38% (score 2) (P<0.001). Median RFS exhibited a stepwise decrease corresponding to increasing CTC-ALRI scores: not reached in the score 0 group, 39.2 months in the score 1 group, and 9.2 months in the score 2 group (log-rank P<0.001). Both Cox regression and logistic regression analyses confirmed the CTC-ALRI score as an independent predictor of RFS and HCC recurrence. The CTC-ALRI score demonstrated excellent predictive performance for both intrahepatic and extrahepatic recurrence, as well as for early and late recurrence. Furthermore, the CTC-ALRI score outperformed conventional factors (AFP, tumor size) and established models (BCLC, TNM, ERASL, SSCLIP, Korean), with areas under the curve of 0.725 and 0.729 for one- and two-year recurrence prediction. Higher scores correlated significantly with Ki-67 proliferation index (P<0.05), providing biological validation.

**Conclusions:**

The preoperative CTC-ALRI score provides superior predictive accuracy for HCC recurrence following curative resection. This accessible, cost-effective biomarker enables refined risk stratification and may guide personalized surveillance and adjuvant treatment strategies.

## Introduction

Liver cancer, predominantly hepatocellular carcinoma (HCC), ranks as the sixth most common malignancy and the third leading cause of cancer-related mortality worldwide (1). Surgical resection remains the cornerstone of curative treatment for patients with early-stage HCC (2, 3). However, postoperative recurrence remains a major clinical challenge, occurring in approximately 50%–70% of patients within five years after surgery (2, 4), significantly undermining the surgical efficacy and limiting long-term survival outcomes. Notably, early recurrence—defined as recurrence within two years—accounts for nearly 70% of all relapsed cases (5). This early recurrence is associated with distinct genomic landscapes and evolutionary trajectories and is generally indicative of a poorer prognosis compared to late recurrence (6, 7). Therefore, there is a critical need to identify novel and reliable biomarkers for the early detection and accurate prediction of HCC recurrence.

Traditional clinicopathological indicators—such as alpha-fetoprotein (AFP), tumor size, and microvascular invasion (MVI) (8), are widely utilized in clinical practice; however, their predictive performance remains suboptimal. In recent years, advanced radiomics (9) and genomics-based (10, 11) approaches have demonstrated improved accuracy in predicting postoperative recurrence of HCC. For instance, Zhao et al. developed a radiomics signature based on contrast-enhanced MRI that achieved an AUC of 0.917 for predicting early recurrence (12), while Cheng et al. constructed a lactylation-related gene signature with promising prognostic discrimination in HCC (13). Nonetheless, despite their statistical performance, neither approach has been widely adopted in routine clinical practice. Radiomics models require specialized imaging acquisition protocols, dedicated analytical software, and significant radiologist expertise, making them difficult to implement outside of high-volume academic centers. As highlighted by Traverso et al., radiomic features are inherently susceptible to variations in image acquisition and reconstruction parameters, preprocessing strategies, and scanner-related heterogeneity, collectively imposing substantial constraints on their reproducibility and generalizability in multi-center and cross-platform validation settings (14). Genomic and transcriptomic profiling involves complex laboratory workflows, prolonged turnaround times, and considerable financial burden—barriers that are particularly prohibitive in resource-limited settings where HCC burden is disproportionately high. Chawla et al. (15) reported that comprehensive genomic profiling(CGP) carries an average cost of several thousand euros per case, and data from nationwide multi-center programs indicate that the median report turnaround time for standardized CGP panels is approximately four weeks (16). In the perioperative context—where clinical decision-making windows are frequently confined to a matter of days—such prohibitive costs and protracted turnaround times critically undermine the feasibility and practical utility of CGP in routine oncological care. Consequently, a substantial translational gap persists between prognostic innovation and bedside implementation.

In contrast, blood-derived biomarker models-obtained through routine clinical laboratory tests offer several compelling advantages: they are minimally invasive, cost-effective, widely accessible, highly reproducible, and objective. Liquid biopsy-derived markers and systemic immune-inflammation indices constitute the most prevalent and well-established blood-derived biomarkers in clinical practice. Liquid biopsy has emerged as a powerful, non-invasive tool in providing real-time tumor information in cancer patients. Circulating tumor cells (CTCs), as an indispensable liquid biopsy classifier, hold significant value for cancer screening and prognosis prediction. A growing body of evidence suggests that elevated CTC counts are strongly associated with increased risk of HCC recurrence (17, 18). Simultaneously, systemic immune-inflammation markers such as the neutrophil-to-lymphocyte ratio (NLR), platelet-to-lymphocyte ratio (PLR), lymphocyte-monocyte ratio (LMR), systemic immune inflammation index (SII), and pan-immune-inflammation value (PIV), have demonstrated prognostic utility in predicting HCC recurrence (19, 20). Of note, the aspartate aminotransferase-to-lymphocyte ratio index (ALRI) has emerged as a novel composite biomarker integrating liver function and systemic inflammation (21).Elevated ALRI levels are associated with inferior prognosis in patients with resectable HCC (22, 23). Despite the individual prognostic value of these biomarkers, most existing studies have evaluated them in isolation. Comprehensive models that integrate multiple complementary blood-based parameters-particularly combining CTCs with systemic immune-inflammation indices- remain conspicuously lacking.

Among the broad spectrum of systemic immune-inflammation markers, ALRI occupies a mechanistically privileged position: whereas conventional indices such as NLR and SII capture only a single dimension of host immune-inflammatory dysregulation, ALRI simultaneously incorporates aspartate aminotransferase—a surrogate of tumor-related hepatocellular injury—and lymphocyte count—a reflection of host immune competence—thereby encompassing two biologically distinct yet interrelated axes of the tumor-host interaction within a single, readily calculable parameter. This mechanistic breadth renders ALRI particularly well-suited for integration with CTCs, whose enumeration directly quantifies the tumor dissemination axis that inflammatory indices alone cannot capture. To the best of our knowledge, no published study has simultaneously integrated CTCs and ALRI, into a composite scoring system for predicting postoperative recurrence in HCC. This gap is particularly noteworthy given that prior blood-based prognostic studies have largely evaluated these markers in isolation. CTC-based studies by Lu et al. (24) and Wang et al. (18) established the prognostic relevance of circulating tumor cells but did not incorporate indices of hepatic inflammation or immune status. Conversely, ALRI-focused investigations by Jin et al. (22) and Peng et al. (23) demonstrated independent prognostic value but failed to account for tumor dissemination as reflected by CTCs. Models incorporating conventional inflammatory indices—such as those reported by Huang et al. (25) and Minici et al. (26), employing combinations of PNI with SII and LMR with NLR, respectively—reflect systemic immune-inflammatory dysregulation but inherently fail to quantify viable circulating tumor cells or capture tumor-induced hepatocellular injury. The CTC-ALRI score proposed herein therefore addresses each of these limitations simultaneously, integrating complementary biological information from two mechanistically distinct yet interrelated domains within a single, clinically accessible framework.

Therefore, this study aimed to systematically evaluate the prognostic value of CTCs and ALRI in patients undergoing curative resection for HCC, and to develop a novel composite scoring system that integrates these complementary biomarkers to enhance the prediction of postoperative recurrence. Critically, this scoring system relies exclusively on parameters obtainable through routine clinical laboratory testing, conferring broad applicability across healthcare settings with varying levels of resource availability. As such, it holds substantial translational potential to bridge the longstanding divide between prognostic innovation and real-world clinical implementation in the management of HCC.

## Materials and methods

### Study population

This retrospective study enrolled HCC patients who underwent curative resection at The Fourth Hospital of Hebei Medical University between January 2019 and July 2023. Inclusion criteria were: (1) age ≥18 years; (2) histopathologically confirmed primary HCC; (3) curative resection performed; (4) availability of preoperative clinical data and CTC detection results; and (5) complete medical records and follow-up data. Patients were excluded if they met any of the following criteria: (1) concomitant malignancies; (2) severe cardiac, pulmonary, or renal dysfunction; (3) acute infection or autoimmune disorder exacerbation within one month before surgery; and (4) incomplete preoperative data. Demographic, laboratory, pathological, and treatment-related data were retrospectively extracted from electronic medical records. Postoperative tumor-node-metastasis (TNM) staging was determined according to the eighth edition of the American Joint Committee on Cancer (AJCC) staging manual (27). This study received approval from the Ethical Committee of the Fourth Hospital of Hebei Medical University (certificate no. 2024KS003) and conducted in accordance with the Declaration of Helsinki.

### CTCs detection

CTCs were detected using the Cyttel method (Cyttel, Jiangsu, China), employing immunomagnetic bead negative enrichment combined with immunofluorescence *in situ* hybridization (imFISH) technology (28). 5 ml peripheral blood sample of HCC patients was collected and assayed within 1 week before surgery. The detection protocol consisted of two steps: First, leukocytes were depleted through red blood cell lysis and immunomagnetic bead separation, enriching for rare circulating cells. Second, the enriched cells were characterized using CD45-negative selection combined with chromosome 8 and 17 imFISH probes and DAPI staining to identify aneuploid cells. This approach circumvents limitations associated with epithelial-mesenchymal transition-related downregulation of epithelial cell adhesion molecules, thereby enhancing CTC detection sensitivity. All 160 patients underwent CTC testing. CTC counts ≥1 were defined as CTC-positive, while CTC counts <1 were classified as CTC-negative.

### ALRI and other blood-derived biomarkers

ALRI was calculated as the ratio of aspartate aminotransferase (AST) level to lymphocyte count. Other inflammation- and nutrition-related indices were calculated using the following formulas: NLR= neutrophil count/lymphocyte count; PLR = platelet count/lymphocyte count; PIV= (neutrophil count × platelet count × monocyte count)/lymphocyte count; LMR= lymphocyte count/monocyte count; SII = (platelet count × neutrophil count)/lymphocyte count; PNI = serum albumin (g/L) + 5 × total lymphocyte count (10^9^/L).

### Definition of preoperative CTC-ALRI score

The CTC-ALRI score was constructed based on preoperative CTC status and ALRI levels as follows: patients with both CTC-positive (CTC count ≥1) and ALRI-high (ALRI ≥18) were assigned a score of 2; patients with either CTC-positive or ALRI-high (but not both) were assigned a score of 1; patients with both CTC-negative (CTC count <1) and ALRI-low (ALRI <18) were assigned a score of 0.

### Scoring systems and prognostic models

The early recurrence after surgery for liver tumor (ERASL) model was calculated according to the following formula (29): ERASL-pre score = 0.818 x Gender (0: Female, 1: Male) + 0.447 x Albumin-Bilirubin (ALBI) grade (0: Grade 1; 1: Grade 2 or 3) + 0.100 x ln(Serum AFP in µg/L) + 0.580 x ln(Tumor size in cm) + 0.492 x Tumor number (0: Single; 1: Two or three; 2: Four or more); ERASL-post score = 0.677 x Gender (0: Female, 1: Male) + 0.458 x Albumin-Bilirubin (ALBI) grade (0: Grade 1; 1: Grade 2 or 3) + 0.661 x microvascular invasion (0: no, 1: yes) + 0.082 x ln(Serum AFP in µg/L) + 0.451 x ln(Tumor size in cm) + 0.379 x Tumor number (0: Single; 1: Two or three; 2: Four or more); Korean model, which included gender, platelet count, serum albumin, tumor volume, and microvascular invasion was assessed according to the standard criteria (30). The SSCLIP scoring system was assessed in accordance with standardized protocols (31), incorporating variables such as CTP stage, tumor morphology, AFP levels, portal vein thrombosis, microvascular invasion, age, ALB, and prothrombin activity. In addition, the AJCC TNM stage (27) and Barcelona Clinic Liver Cancer (BCLC) stage (32) were determined according to their respective guidelines.

### Surveillance and follow-up strategy

Postoperative surveillance comprised physical examination and laboratory assessments, including tumor markers such as alpha-fetoprotein (AFP), conducted every 3 months during the first 2 years, every 6 months from years 3 to 5, and annually thereafter. Abdominal enhanced computed tomography or magnetic resonance imaging was performed at least annually for a minimum of 3 years. Additional imaging modalities, such as positron emission tomography and SPECT bone scan, were utilized when clinically indicated to confirm recurrence. Follow-up data were obtained from medical records or telephone interviews with patients or their families. All patients were followed until August 1, 2025, or death, whichever occurred first. Recurrence-free survival (RFS) was defined as the interval from the date of resection to the date of tumor recurrence, distant metastasis, or last follow-up.

### Ki-67 assessment

Ki-67 proliferation index data were obtained from pathology reports. The Ki-67 index was reported as the percentage of tumor cells demonstrating positive nuclear staining on immunohistochemical (IHC) analysis.

### Statistical analysis

Continuous variables were compared using Student’s t-test or Mann-Whitney U test/Kruskal-Wallis test and presented as mean ± standard error (SE) or median with range, as appropriate. Categorical variables were compared using Pearson’s chi-square test or Fisher’s exact test. Cumulative RFS probabilities were estimated using the Kaplan-Meier method, and survival differences among CTC-ALRI score groups were assessed using the log-rank test. Variables significantly associated with RFS in univariate analysis were entered into multivariate Cox proportional hazards regression models to identify independent prognostic factors. Hazard ratios (HRs) with corresponding 95% confidence intervals (CIs) were calculated. Fine-Gray competing risks models were employed to estimate the cumulative incidence functions of intrahepatic and extrahepatic recurrence. For the analysis of intrahepatic recurrence, the primary event was defined as the first occurrence of isolated intrahepatic recurrence, with extrahepatic recurrence, simultaneous intrahepatic and extrahepatic recurrence, and death without prior recurrence treated as competing events. Conversely, for the analysis of extrahepatic recurrence, the primary event was defined as the first occurrence of isolated extrahepatic recurrence, with intrahepatic recurrence, simultaneous intrahepatic and extrahepatic recurrence, and death without prior recurrence treated as competing events. Patients who remained alive and recurrence-free at the last follow-up were treated as censored observations. A landmark analysis at 24 months was performed to separately evaluate the associations of CTC, ALRI, and CTC-ALRI score with early (≤24 months) and late (>24 months) recurrence (5). For the early recurrence analysis, all patients were included with follow-up administratively censored at 24 months; patients who experienced recurrence or died prior to 24 months were counted as events or competing events, respectively, within this window. For the late recurrence analysis, the risk set was restricted to patients who were both alive and recurrence-free at the 24-month landmark time point; patients who had experienced recurrence or death prior to 24 months were excluded from this analysis, and the time origin was reset to the landmark point to avoid guarantee-time bias inherent in naive subgroup analyses by time of recurrence. Receiver operating characteristic (ROC) curve analysis was performed to compare the prognostic performance of independent predictors, and areas under the curve (AUCs) were calculated. The time-dependent concordance index (C-index) was computed to assess the discriminative ability of the predictive models. Two-sided P values <0.05 were considered statistically significant. Statistical analyses were performed using SPSS software (version 22.0; SPSS Inc., Chicago, IL, USA), with data visualization conducted using R software (version 4.2.1) and GraphPad Prism 10.0 (GraphPad Software, San Diego, CA, USA).

## Results

### Patient characteristics

A total of 160 HCC patients meeting the inclusion criteria were enrolled in this study. The demographic and clinicopathological characteristics are summarized in **Table 1**. The median age was 60 years, with 51.25% of patients younger than 60 years. The cohort comprised 123 males (76.88%) and 37 females (23.12%). Most patients had underlying hepatitis (93.12%) and cirrhosis (77.50%). Tumor diameter exceeded 5 cm in 53.75% of cases, and 85.63% presented with solitary lesions. The majority of patients had AFP levels <400 ng/mL (70.63%) and were classified as albumin-bilirubin (ALBI) grade 1 (60.63%). Furthermore, most patients showed no evidence of microvascular invasion (MVI, 76.88%) and were categorized as TNM stage I-II (82.50%) and BCLC stage 0/A (78.13%).

**Table 1:** Baseline clinicopathologic characteristics and their association with CTC-ALRI score in 160 HCC patients.

Characteristics	Frequency (n=160)	CTC-ALRI score			*P* value
		Score0 (n=25)	Score 1 (n=71)	Score 2 (n=64)	
Age					0.903
<60	82 (51.25%)	13	35	34	
≥60	78 (48.75%)	12	36	30	
Gender					0.954
Female	37 (23.13%)	6	17	14	
Male	123 (76.87%)	19	54	50	
Hepatitis B/C					0.069
Absent	11 (6.88%)	4	2	5	
Present	149 (93.12%)	21	69	59	
Cirrhosis					0.201
Absent	36 (22.50%)	9	15	12	
Present	124 (77.50)	16	56	52	
Tumor size					0.297
<5cm	86 (53.75%)	17	36	33	
≥5cm	74 (46.25%)	8	35	31	
Tumor number					0.905
Solitary	137 (85.63%)	22	61	54	
Multiple	23 (14.37%)	3	10	10	
AST (U/L)	31.5 (24.3, 52.1)	23.2 (19.6, 27.5)	32.6 (24.3, 53.7)	39.5 (26.3, 61.0)	0.000
ALT (U/L)	29.7 (20.9, 49.3)	24.1 (19.3, 34.8)	29.3 (21.8, 44.6)	34.7 (21.9, 64.5)	0.020
ALBI grade					0.213
1	97 (60.63%)	18	45	34	
2	63 (39.37%)	7	26	30	
AFP					0.004
<400ng/ml	113 (70.63%)	23	53	37	
≥400ng/ml	47 (29.37%)	2	18	27	
MVI					0.085
Absent	123 (76.88%)	23	50	50	
Present	37 (23.12%)	2	21	14	
TNM stage					0.378
I/II	132 (82.50%)	23	58	51	
III	28 (17.50%)	2	13	13	
BCLC stage					0.577
0/A	125 (78.13%)	21	53	51	
B/C	35 (21.87%)	4	18	13	

To determine optimal cutoff values for blood-derived biomarkers predicting postoperative recurrence, ROC curve analysis was performed. The optimal thresholds were identified as follows: NLR 2.244, PLR 125.98, PIV 93.27, LMR 3.74, SII 333.66, PNI 52.30, and ALRI 17.56 (**Supplementary Figure 1**). CTC analysis identified 86 patients (53.75%) as CTC-positive (≥1) and 74 patients (46.25%) as CTC-negative (<1).

### Univariate and multivariate Cox regression analysis

**Figure 1:**
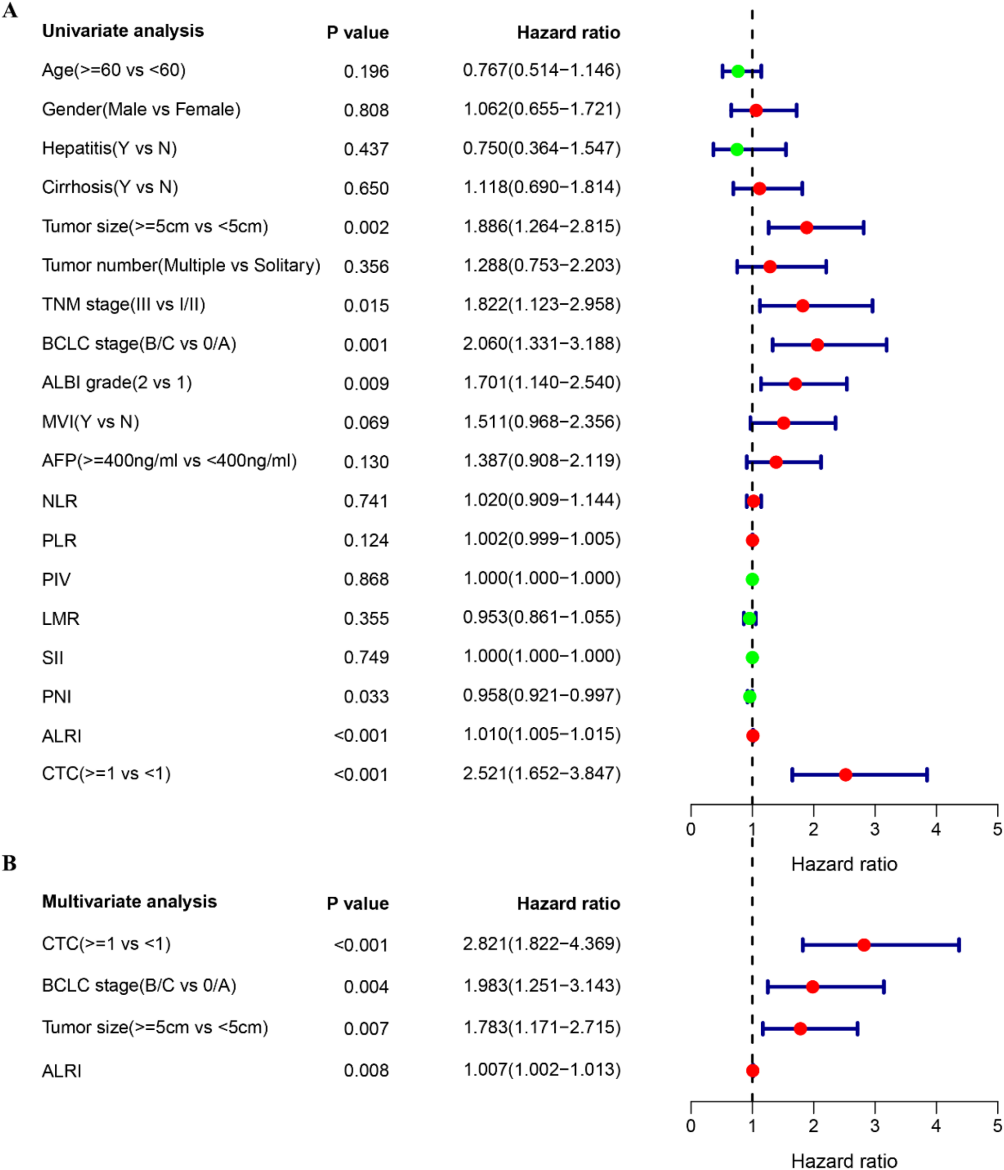
Univariate and multivariate Cox regression of factors predicting RFS in HCC patients. **(A)** Forest plot of the univariate Cox regression of factors associated with RFS in HCC patients; **(B)** Forest plot of the multivariate Cox regression of factors associated with RFS in HCC patients.

**Figure 2:**
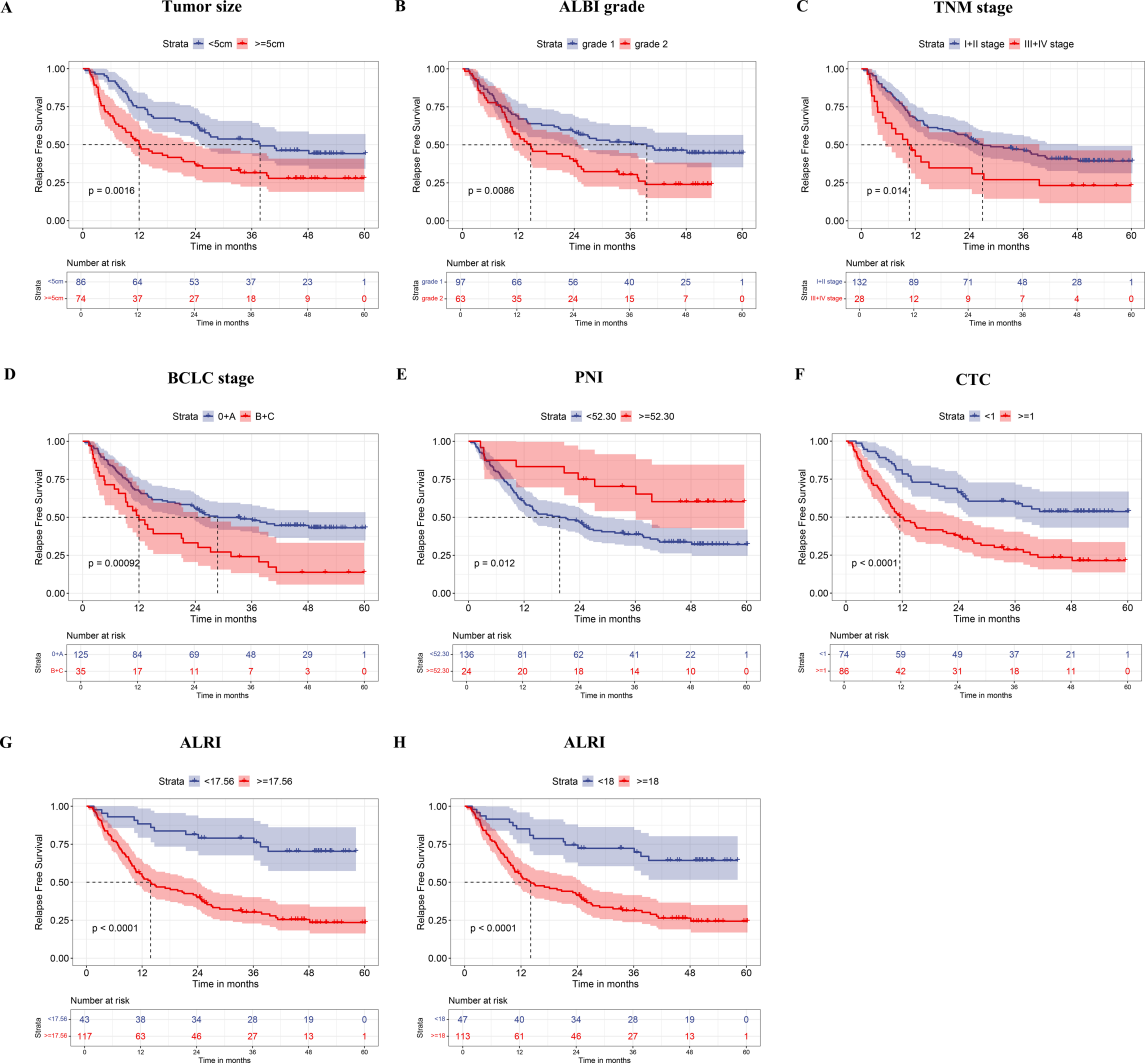
Kaplan-Meier analysis of factors associated with recurrence-free survival. Survival curves stratified by **(A)** tumor size, **(B)** ALBI grade, **(C)** TNM stage, **(D)** BCLC stage, **(E)** prognostic nutritional index (PNI), **(F)** CTC status, **(G)** ALRI (cut-off 17.56), and **(H)** ALRI (cut-off 18).

**Figure 3:**
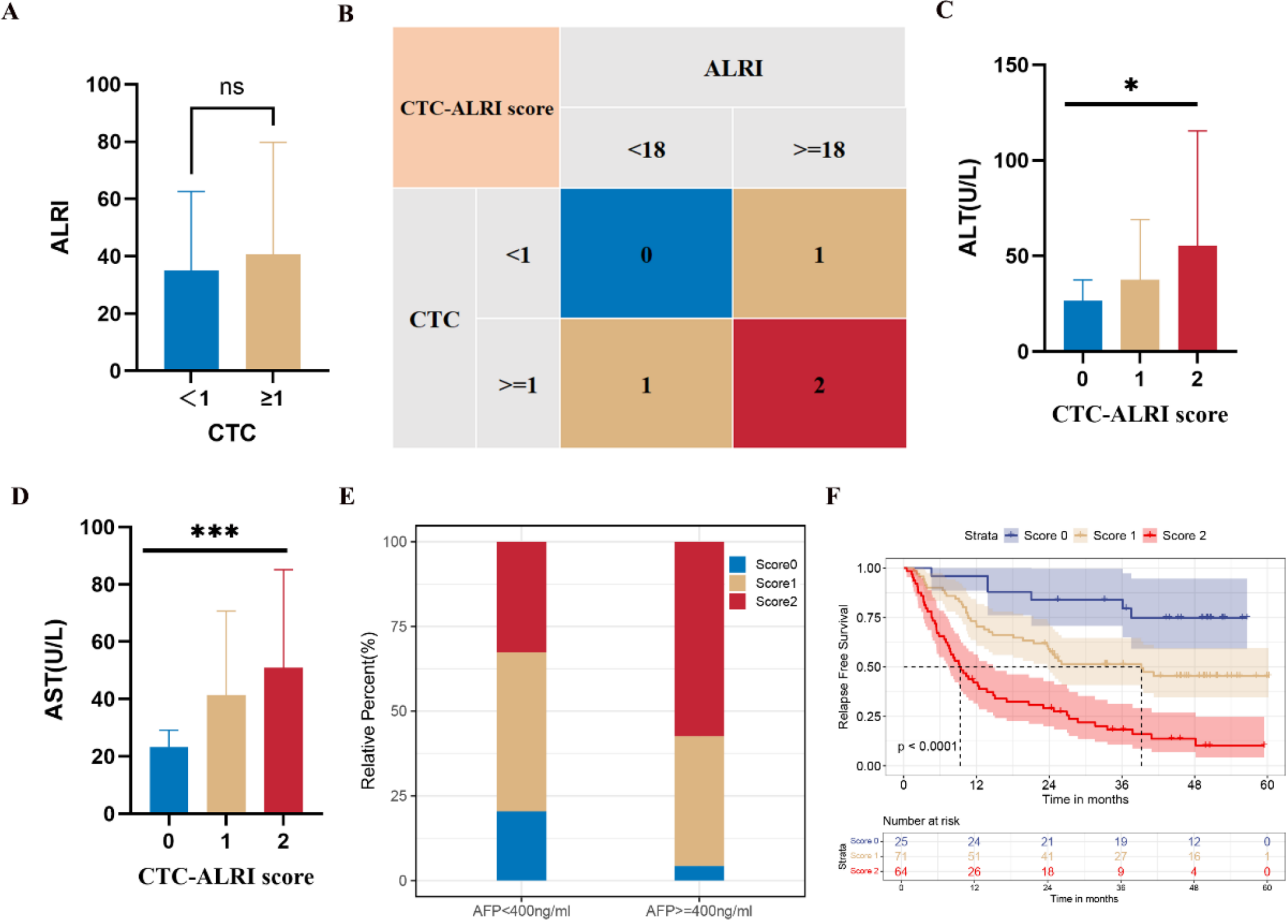
Definition of the CTC-ALRI score and its association with clinicopathological features. **(A)** Correlation between preoperative ALRI and CTC status. **(B)** Definition of CTC-ALRI score. **(C-E)** Associations of the CTC-ALRI score with ALT, AST, and AFP levels. **(F)** Kaplan-Meier analysis of RFS according to CTC-ALRI score in HCC patients following curative resection. *P < 0.05, ***P < 0.001.

To identify prognostic factors associated with RFS in HCC patients who underwent curative resection, univariate and multivariate Cox proportional hazards regression analyses were performed. The median RFS for the entire cohort was 24.57 months. On univariate analysis, established clinicopathological prognostic factors—including tumor size, BCLC stage, TNM stage, and ALBI grade—were each significantly associated with RFS (**Figure 1**; all P < 0.05). Notably, blood-derived biomarkers including PNI, CTC status, and ALRI also demonstrated significant associations with RFS on univariate analysis (**Figure 1**; all P < 0.05). Kaplan-Meier survival analysis further revealed that patients with tumor diameter ≥5 cm, ALBI grade 2, advanced TNM and BCLC stages, PNI <52.30, CTC count ≥1, and ALRI ≥17.56 exhibited significantly shorter RFS compared with their respective counterparts (**Figures 2A–G**). On multivariate analysis, CTC status (P < 0.001) and ALRI (P = 0.008) were identified as independent prognostic factors for RFS (**Figure 1**). Although ALRI levels tended to be higher in the CTC-positive group, this difference did not reach statistical significance (P = 0.460) (**Figure 3A**), suggesting that these two biomarkers may reflect complementary rather than overlapping biological processes.

### Correlation between preoperative CTC-ALRI score and clinicopathological characteristics

Given that both CTC status and ALRI were identified as independent predictors of recurrence, we developed a composite CTC-ALRI scoring system based on these two variables. As the ALRI cutoff value of 17.56 was empirically derived from ROC curve analysis, a subsequent sensitivity analysis was performed using a clinically intuitive cutoff of 18—which more closely approximates the threshold of 18.73 reported in a prior study (33)—to assess the robustness of the primary findings. This alternative cutoff demonstrated comparable discriminatory capacity for RFS stratification in HCC patients following curative resection (**Figure 2H**), thereby corroborating the stability and clinical applicability of the ALRI threshold established in the present study. Patients with CTC <1 and ALRI <18 were assigned a score of 0; those with either CTC ≥1 or ALRI ≥18 (but not both) were assigned a score of 1; and patients with both CTC ≥1 and ALRI ≥18 were assigned a score of 2 (**Figure 3B**). This stratification yielded 25 (15.63%), 71 (44.37%), and 64 patients (40.00%) in the score 0, 1, and 2 groups, respectively (**Table 1**).

The associations between preoperative CTC-ALRI scores and clinicopathological characteristics are presented in **Table 1**. The CTC-ALRI score correlated significantly with alanine aminotransferase (ALT) level (P = 0.020), AST level (P < 0.001), and AFP level (P = 0.004) (**Figures 3C–E**), indicating its association with hepatic dysfunction and tumor burden. Notably, the median RFS decreased dramatically across CTC-ALRI score groups: not reached for score 0, 39.2 months for score 1, and 9.2 months for score 2 (P < 0.001) (**Figure 3F**).

### The CTC-ALRI score predicts postoperative recurrence in HCC patients

**Figure 4:**
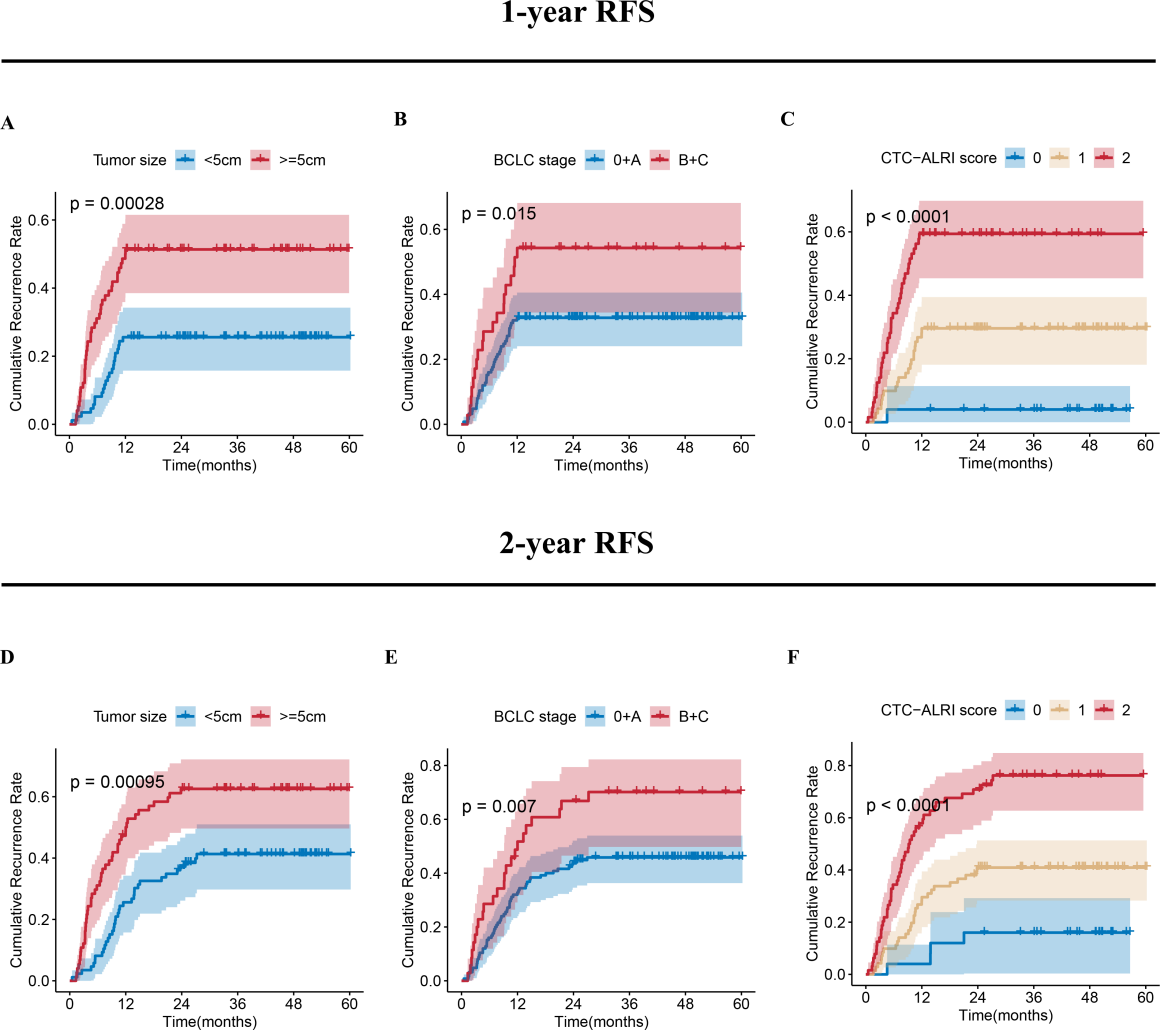
Cumulative recurrence rates for tumor size, BCLC stage, and CTC-ALRI score. **(A-C)** One-year cumulative recurrence rates stratified by tumor size, BCLC stage, and CTC-ALRI score. **(D-F)** Two-year cumulative recurrence rates stratified by tumor size, BCLC stage, and CTC-ALRI score.

**Figure 5:**
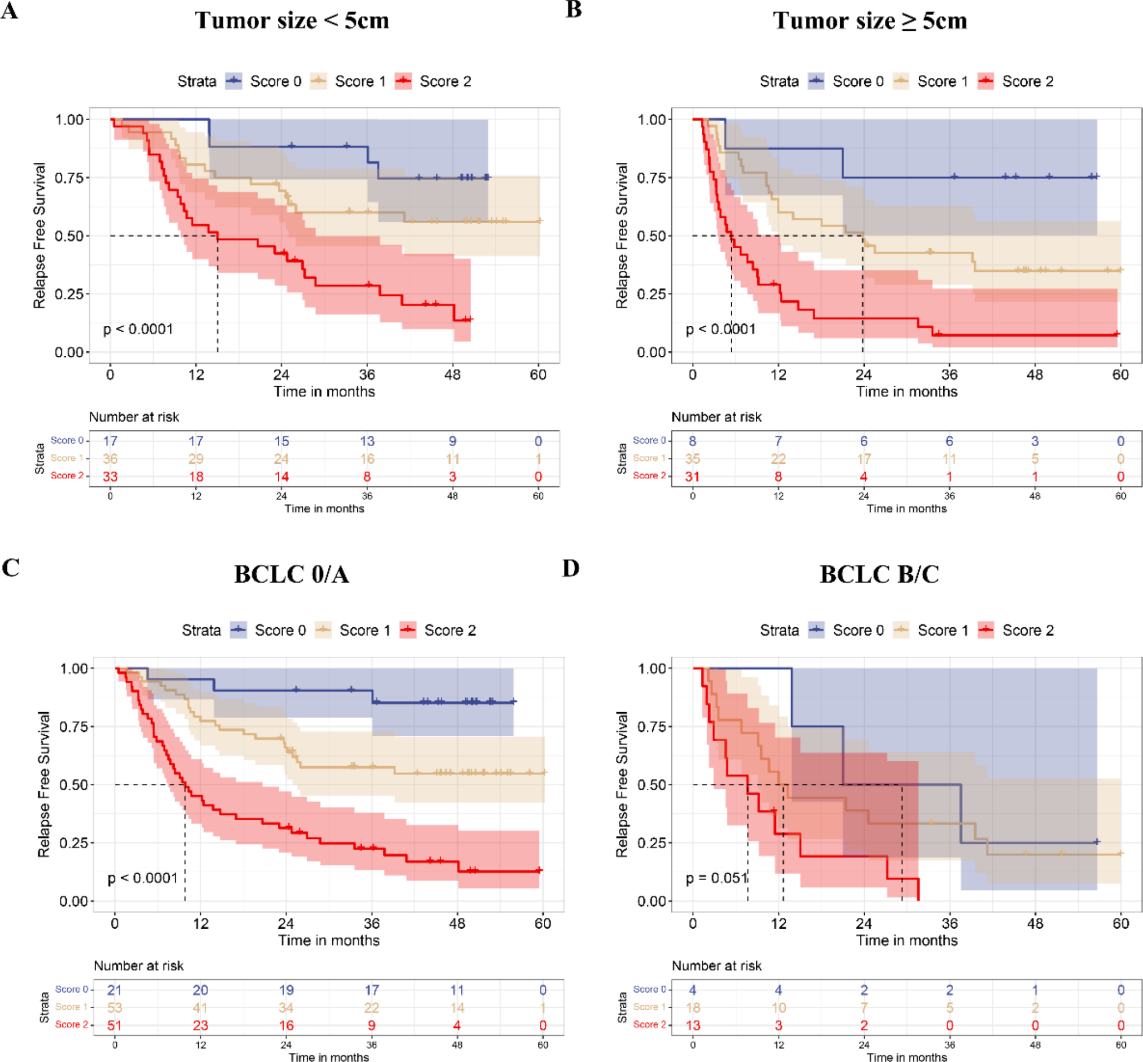
Recurrence-free survival according to CTC-ALRI score stratified by clinical features. **(A, B)** Kaplan-Meier curves stratified by tumor size. **(C, D)** Kaplan-Meier curves stratified by BCLC stage.

To validate the predictive value of the CTC-ALRI score for postoperative RFS in HCC, this scoring system was incorporated into multivariate Cox proportional hazards regression analysis, which identified tumor size, BCLC stage, and CTC-ALRI score as independent predictors of postoperative RFS (**Table 2**). Further analysis demonstrated that tumor size, BCLC stage and CTC-ALRI score exhibited satisfactory discriminative ability for predicting 1-year (**Figures 4A–C**) and 2-year recurrence (**Figures 4D–F**). Stratified analyses by tumor size and BCLC stage revealed that higher CTC-ALRI scores were consistently associated with shorter RFS across all subgroups (**Figures 5A–D**). Specifically, among patients with tumor size <5 cm and those with tumor size ≥5 cm, significantly shorter RFS was observed with increasing CTC-ALRI scores (both P < 0.0001). Similarly, in the BCLC 0/A subgroup, the CTC-ALRI score demonstrated strong prognostic discrimination (P < 0.0001), and a consistent trend toward shorter RFS with higher scores was also observed in the BCLC B/C subgroup (P = 0.051), underscoring the robust prognostic value of this scoring system across distinct patient subgroups. The statistically significant variables identified in the multivariate Cox model were subsequently entered into multivariate logistic regression analysis, which confirmed that the CTC-ALRI score remained an independent predictor of postoperative recurrence in HCC (**Table 3**), further substantiating the robustness and consistency of its prognostic utility across complementary analytical frameworks.

**Table 2:** Cox regression analysis for predictors of recurrence-free survival of HCC patients.

Variable	Univariate analysis			Multivariate analysis		
	HR	95%CI	*P*	HR	95%CI	*P*
Tumor size (cm) (≥5 vs <5)	1.886	1.264-2.814	0.002	1.817	1.195-2.761	0.005
ALBI (Grade 2 vs 1)	1.701	1.139-2.539	0.009			
TNM stage (III+IV vs I+II)	1.823	1.123-2.958	0.015			
BCLC stage (B/C vs 0/A)	2.060	1.331-3.188	0.001	1.865	1.186-2.932	0.007
PNI(≥52.30 vs <52.30)	0.427	0.215-0.849	0.015			
ALRI (≥18 vs <18)	3.156	1.840-5.413	0.000			
CTC status (≥1 vs <1)	2.520	1.651-3.847	0.000			
CTC-ALRI score (1)	2.880	1.214-6.831	0.016	2.524	1.061-6.003	0.036
CTC-ALRI score (2)	7.579	3.240-17.730	0.000	7.684	3.278-18.008	0.000

**Table 3:** Logistic regression analysis for predictors of HCC recurrence.

Variable	β	SE	Wald	OR	(95%CI)	*P*
BCLC stage (B/C vs 0/A)	1.699	0.527	10.039	5.306	1.890-14.895	0.002
CTC-ALRI score (1)	1.206	0.553	4.747	3.340	1.129-9.882	0.029
CTC-ALRI score (2)	2.979	0.611	23.798	19.664	5.942-65.078	0.000

**Figure 6:**
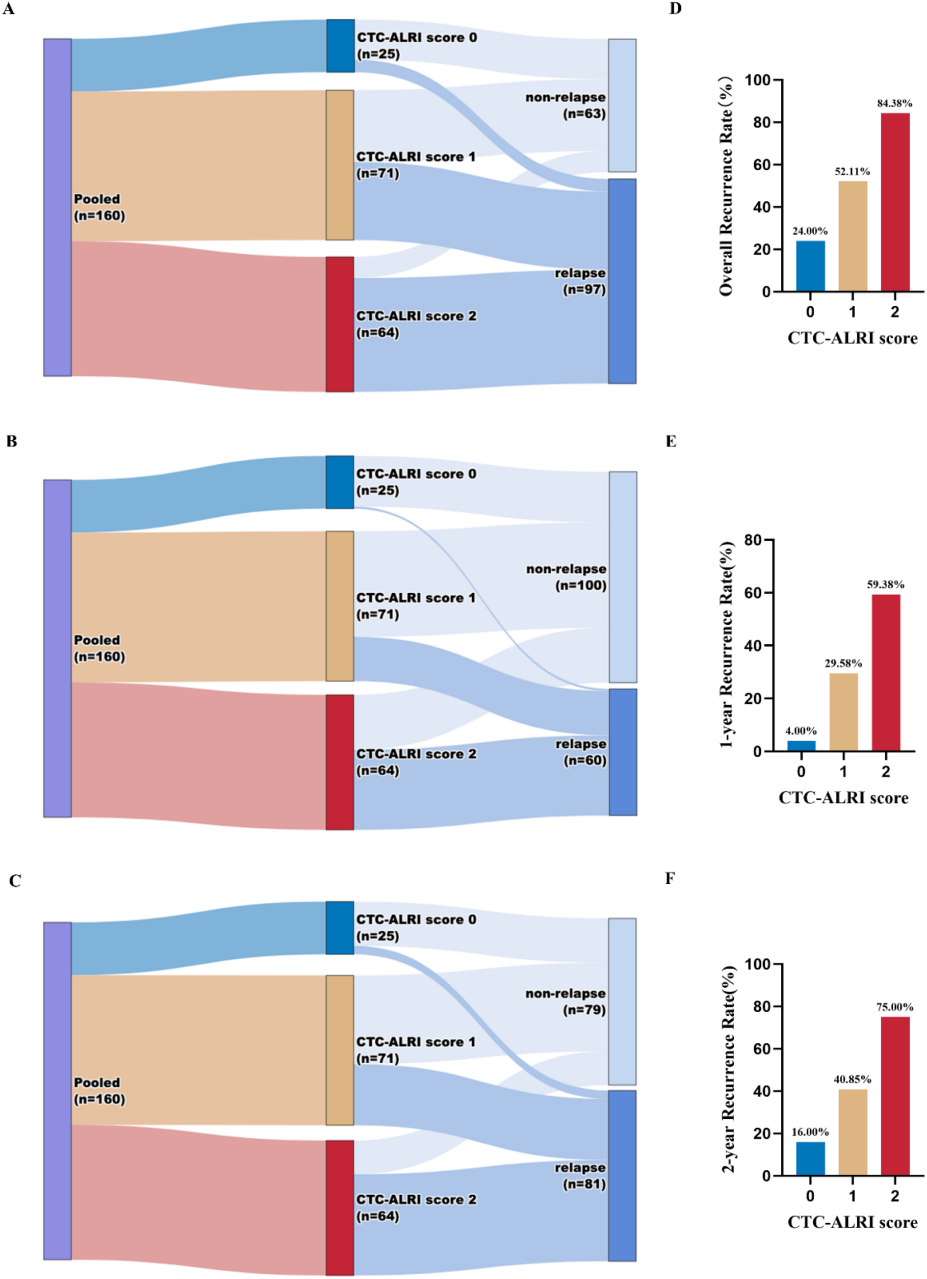
Distribution of recurrence outcomes and recurrence rates stratified by CTC-ALRI score. **(A-C)** Sankey diagrams illustrating the distribution of overall **(A)**, 1-year **(B)**, and 2-year **(C)** recurrence outcomes across CTC-ALRI score groups (score 0, n=25; score 1, n=71; score 2, n=64) in the pooled cohort (n=160). **(D-F)** Bar charts depicting the overall **(D)**, 1-year **(E)**, and 2-year **(F)** recurrence rates according to CTC-ALRI score.

We next evaluated the association between the CTC-ALRI score and postoperative recurrence. At the last follow-up, 97 patients (60.63%) had experienced disease recurrence. The recurrence rates increased progressively with higher CTC-ALRI scores: 24.00% for score 0, 52.11% for score 1, and 84.38% for score 2 (P < 0.001) (**Figures 6A, D**). Similarly, the 1-year recurrence rates were 4.00%, 29.58%, and 59.38% for scores 0, 1, and 2, respectively, while the corresponding 2-year recurrence rates were 16.00%, 40.85%, and 75.00% (**Figures 6B, C, E, F**, all P < 0.001).

### Predictive value of the CTC-ALRI score for different recurrence patterns and recurrence timing

**Figure 7:**
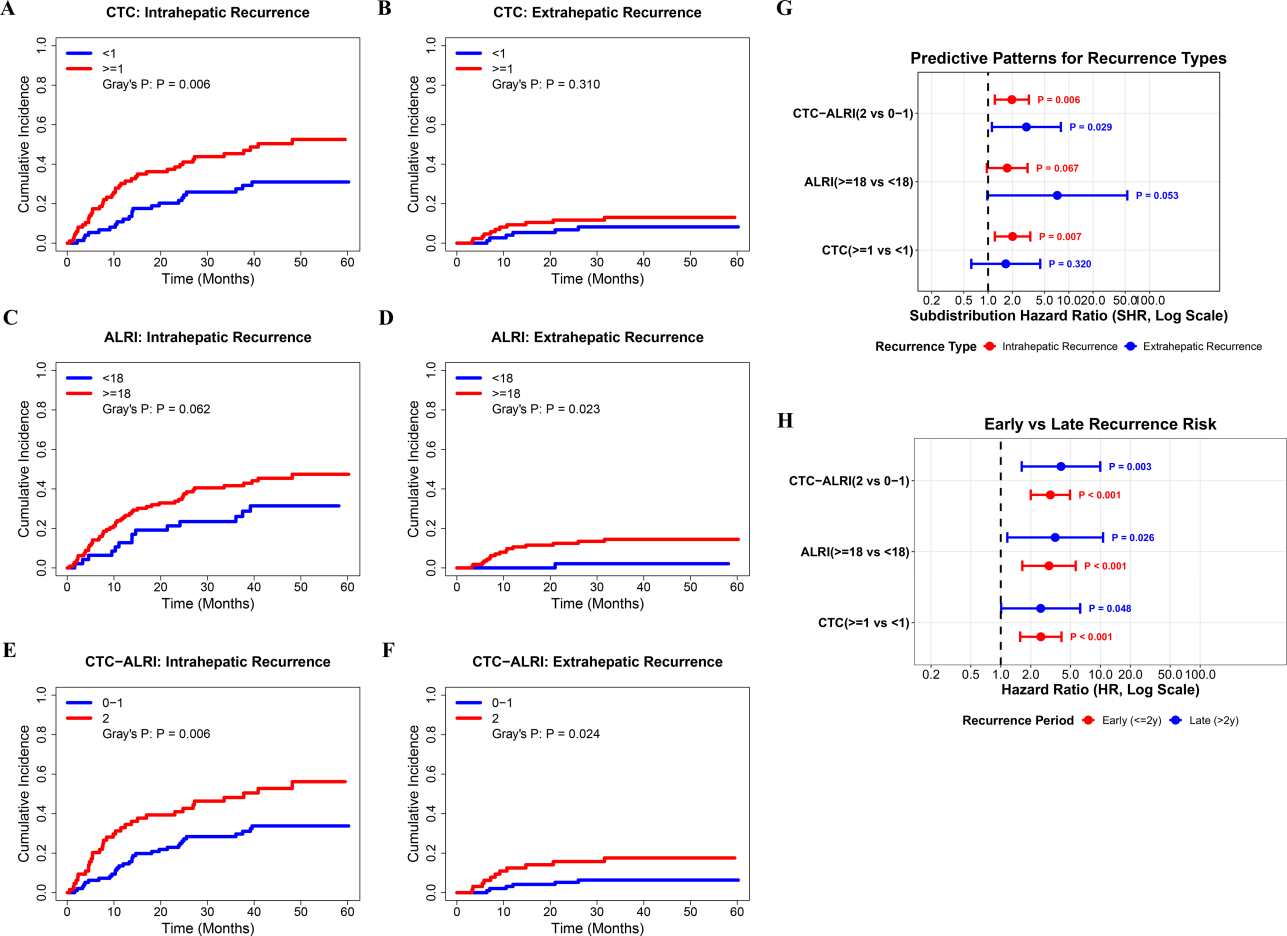
Competing risks analysis and landmark analysis of CTC, ALRI, and CTC-ALRI score for predicting recurrence patterns and temporal recurrence risk in HCC patients following curative resection. **(A, B)** Cumulative incidence functions of intrahepatic recurrence **(A)** and extrahepatic recurrence **(B)** stratified by CTC status (CTC <1 vs. ≥1), estimated using Fine-Gray competing risks regression analysis. **(C, D)** Cumulative incidence functions of intrahepatic recurrence **(C)** and extrahepatic recurrence **(D)** stratified by ALRI level (ALRI <18 vs. ≥18). **(E, F)** Cumulative incidence functions of intrahepatic recurrence **(E)** and extrahepatic recurrence **(F)** stratified by CTC-ALRI score (score 0–1 vs. 2). Gray’s test P-values are indicated within each panel. **(G)** Forest plot depicting subdistribution hazard ratios (SHRs) with 95% confidence intervals derived from Fine-Gray competing risks regression, illustrating the differential predictive patterns of CTC-ALRI score, ALRI, and CTC status for intrahepatic recurrence (red circles) and extrahepatic recurrence (blue circles). **(H)** Forest plot depicting hazard ratios (HRs) with 95% confidence intervals derived from landmark analysis, comparing the predictive performance of CTC-ALRI score, ALRI, and CTC status for early recurrence (≤2 years, red circles) and late recurrence (>2 years, blue circles).

To further characterize the predictive specificity of CTC, ALRI, and the CTC-ALRI score for distinct recurrence patterns, Fine-Gray competing risks regression analysis was performed, with results summarized in **Figure 7**. Fine-Gray competing risks analysis demonstrated that elevated CTC status (CTC ≥1) was significantly associated with a higher cumulative incidence of intrahepatic recurrence (Gray’s P = 0.006; **Figure 7A**), whereas no significant difference was observed for extrahepatic recurrence between CTC strata (Gray’s P = 0.310; **Figure 7B**). In contrast, elevated ALRI (≥18) did not reach statistical significance for intrahepatic recurrence (Gray’s P = 0.062; **Figure 7C**), but was significantly associated with a markedly higher cumulative incidence of extrahepatic recurrence (Gray’s P = 0.023; **Figure 7D**). The CTC-ALRI score demonstrated significant discriminatory capacity for both intrahepatic recurrence (Gray’s P = 0.006; **Figure 7E**) and extrahepatic recurrence (Gray’s P = 0.024; **Figure 7F**), suggesting that the composite score captures a broader spectrum of recurrence risk than either individual marker alone. The forest plot of subdistribution hazard ratios (SHR) further illustrated the differential predictive patterns of these biomarkers (**Figure 7G**). The CTC-ALRI score (2 vs. 0-1) was independently associated with both intrahepatic recurrence (SHR, P = 0.006) and extrahepatic recurrence (SHR, P = 0.029). ALRI (≥18 vs. <18) was preferentially associated with extrahepatic recurrence (P = 0.053) rather than intrahepatic recurrence (P = 0.067). CTC status (≥1 vs. <1) was significantly associated with intrahepatic recurrence (P = 0.007) but not with extrahepatic recurrence (P = 0.320). These findings suggest that CTCs predominantly predict intrahepatic dissemination, ALRI preferentially predicts extrahepatic spread, and the CTC-ALRI composite score captures both recurrence patterns simultaneously.

With respect to temporal recurrence risk, landmark analysis using a 24-month time point revealed that all three biomarkers were significantly associated with both early (≤2 years) and late (>2 years) recurrence (**Figure 7H**). The CTC-ALRI score demonstrated significant associations with early recurrence (HR, P < 0.001) and late recurrence (HR, P = 0.003). Similarly, ALRI was significantly predictive of both early (P < 0.001) and late recurrence (P = 0.026), as was CTC status for both early (P < 0.001) and late recurrence (P = 0.048). Collectively, these findings indicate that the CTC-ALRI score provides robust and consistent prognostic discrimination across both recurrence phases, underscoring its potential utility for long-term postoperative surveillance stratification.

### Comparison of the predictive value of independent prognostic factors

**Figure 8:**
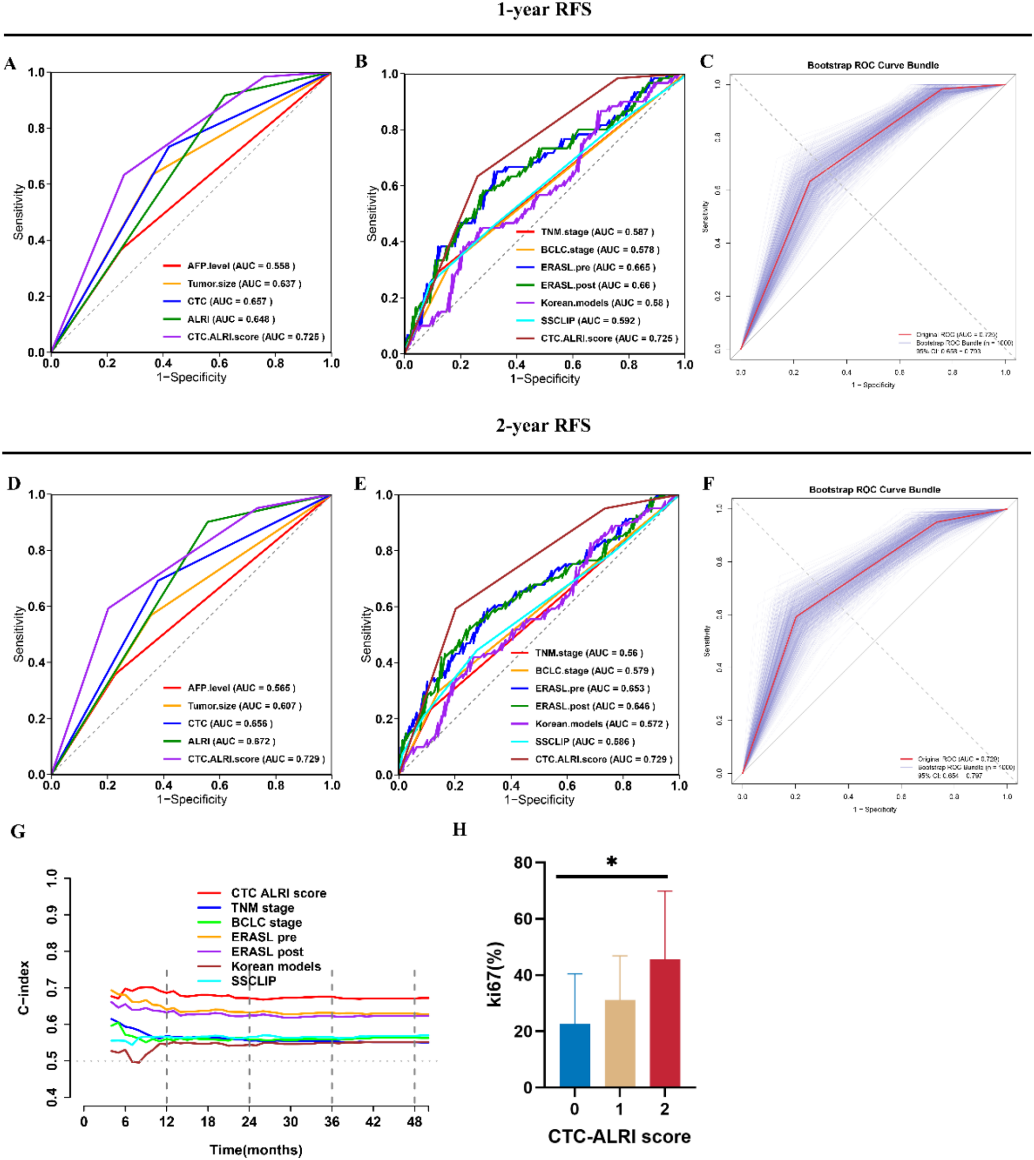
Comparative predictive performance of CTC-ALRI score, conventional biomarkers, and staging systems for postoperative recurrence. **(A, D)** ROC curves comparing different prognostic factors for predicting 1-year and 2-year recurrence. **(B, E)** ROC curves comparing established staging systems for predicting 1-year and 2-year recurrence. **(C, F)** Bootstrap validation of CTC-ALRI score for 1-year and 2-year recurrence prediction. **(G)** Time-dependent C-index of different prognostic models. **(H)** Ki-67 proliferation index stratified by CTC-ALRI score groups. *P < 0.05.

ROC analysis indicated that the preoperative CTC-ALRI score provided higher predictive accuracy for recurrence compared with AFP and tumor size. Furthermore, the combined CTC-ALRI score outperformed either CTC or ALRI alone in predicting 1-year and 2-year recurrence (**Figures 8A, D**). We also compared the predictive performance of CTC-ALRI score with the with other traditional models. The CTC-ALRI score demonstrated superior predictive performance with AUCs of 0.725 and 0.729 for one-year and two-year predictions, respectively, outperforming the ERASL model, Korean model, SSCLIP scoring system, AJCC TNM staging system, and BCLC staging system (**Figures 8B, E**). Bootstrap resampling with 1,000 iterations confirmed the robustness and stability of the CTC-ALRI score (**Figures 8C, F**). Time-dependent C-index analysis further validated the superior discriminative ability of the CTC-ALRI score over other traditional models throughout the follow-up period (**Figure 8G**). Collectively, these findings establish the CTC-ALRI score as a reliable and superior predictor of postoperative recurrence in HCC patients.

### The correlation between the CTC-ALRI score and Ki-67

To further validate the biological relevance of the CTC-ALRI score, we analyzed Ki-67 proliferation index data from 46 patients with available tissue samples (11 patients with score 0, 17 with score 1, and 18 with score 2). The Ki-67 index increased progressively with higher CTC-ALRI scores, with statistically significant differences among the three groups (P < 0.05) (**Figure 8H**). This finding suggests that the CTC-ALRI score reflects tumor proliferative capacity and provides biological validation for its prognostic value.

## Discussion

Despite substantial advances in surgical techniques and perioperative management for HCC, postoperative recurrence remains the major bottleneck in improving patient outcomes (34, 35). Therefore, accurate risk estimation of recurrence is of paramount clinical importance for individualized treatment, management, and surveillance strategies in the postoperative period. Currently, several prediction models have been developed based on recurrence-associated risk factors—including large tumor size, elevated serum AFP levels, vascular invasion, poor differentiation, and specific imaging features (8, 36). However, no consensus has been reached regarding the optimal risk-stratification tool for predicting HCC recurrence. Moreover, these markers inadequately reflect intrinsic tumor biology and demonstrate limited ability to profile the comprehensive landscape of tumor aggressiveness, including variations in systemic immune-inflammatory status and the presence of micrometastatic disease. In the present study, we developed a novel CTC-ALRI score as an integrated biomarker for predicting recurrence in 160 HCC patients undergoing curative resection. To the best of our knowledge, this is the first study demonstrating that the preoperative CTC-ALRI score correlates with tumor progression and serves as an independent predictor of recurrence in HCC patients following curative resection.

ALRI, calculated as the ratio of AST to lymphocyte count, comprehensively reflects the balance between hepatic inflammation and host immune response. ALRI has demonstrated prognostic significance across various solid tumors, including intrahepatic cholangiocarcinoma (37), colorectal cancer (38), gallbladder Cancer (39) and hepatocellular carcinoma (23). Elevated AST levels indicate liver cell injury and inflammation, which may create a microenvironment conducive to tumor cell proliferation and survival. Prior studies have consistently identified serum AST level as one of the clinicopathological variables most closely and independently associated with postoperative recurrence in HCC (40, 41). Conversely, lymphocytes play a crucial role in tumor immune surveillance by inducing cytotoxic cell death and maintaining anti-tumor immunity. Decreased lymphocyte counts may compromise immune-mediated tumor control, thereby facilitating malignant progression. Therefore, ALRI represents a rational composite biomarker integrating liver injury and immunological status to predict poor prognosis in cancer patients. Our study demonstrated that elevated ALRI predicted HCC recurrence and was associated with shorter RFS. Previous studies have established both preoperative (22) and postoperative (42) ALRI as an independent prognostic factor following hepatic resection for HCC. However, the prognostic significance of dynamic ALRI changes in predicting postoperative recurrence and overall survival remains to be elucidated and represents a focus of our ongoing research. Noteworthily, recent investigations have shown that elevated ALRI correlates with poor outcomes in HCC patients undergoing TACE (43, 44) and those receiving palliative treatment (45). Nevertheless, the prognostic value of ALRI in patients with advanced HCC receiving combined targeted therapy and immunotherapy—the current first-line treatment (46), remains to be determined and warrants further investigation.

CTCs, detected in the peripheral blood of cancer patients, were initially recognized for their role in promoting tumor recurrence and distant metastasis (47)and have subsequently been demonstrated to correlate with inferior prognosis across diverse malignancies (48, 49). In HCC, CTCs serve as valuable biomarkers for early diagnosis, prognostic evaluation, detection of minimal residual disease, and assessment of therapeutic response (50, 51). Our study revealed that CTC positivity was significantly associated with postoperative HCC recurrence, particularly early recurrence within one and two years. Furthermore, CTC positivity correlated with significantly shorter RFS following curative resection, consistent with previous reports (24). The biological basis for this association likely involves the shedding of viable tumor cells into circulation, which can establish distant micrometastases and contribute to recurrence even after surgical resection.

Over recent decades, numerous clinicopathological, radiomics, and genomics biomarkers have been identified for predicting HCC recurrence. However, high costs, technical complexity, and limited accessibility have hindered their widespread clinical implementation. Although tissue samples from patients undergoing curative resection are readily obtainable, hematological indices confer several distinct advantages over conventional histological assessment. First, blood-based parameters can be measured preoperatively, whereas tissue-based analyses are necessarily contingent upon postoperative pathological processing; preoperative risk stratification thereby enables timely refinement of surgical strategy and facilitates informed consideration of neoadjuvant therapeutic options. Second, hematological biomarkers are amenable to serial, non-invasive measurement, supporting longitudinal dynamic monitoring throughout postoperative surveillance—an advantage not afforded by tissue-based evaluation, which is inherently limited to a single time point. Third, circulating blood-derived biomarkers may capture systemic tumor burden and host immune status in a manner that reflects the whole-organism biological milieu, whereas histological examination is confined to characterizing the local tumor microenvironment. Therefore, this study systematically evaluated the prognostic value of multiple blood-derived markers—including NLR, PLR, LMR, SII, PIV, PNI, ALRI, and CTC—for postoperative HCC recurrence. Our results demonstrated that PNI, ALRI, and CTC were significantly associated with postoperative recurrence. Specifically, lower PNI and higher ALRI, and CTC levels correlated with shorter RFS, consistent with previous findings (17, 22, 52). Multivariate analysis identified CTC status and ALRI as independent predictors of post-surgical recurrence. Based on these two complementary biomarkers, we constructed a novel CTC-ALRI scoring system and validated its superior predictive performance for postoperative recurrence and its role as an independent predictor of RFS. Importantly, since the CTC-ALRI score is derived from two readily available laboratory parameters obtained through routine blood analysis, it offers the advantages of objectivity, convenience, non-invasiveness, and broad applicability in clinical practice.

Our results demonstrated that the preoperative CTC-ALRI score serves as an independent prognostic biomarker for HCC recurrence. ROC curve analysis revealed that the AUC of the CTC-ALRI score exceeded those of CTC or ALRI alone, providing compelling evidence for its superior predictive performance. This enhanced prognostic value likely stems from the complementary nature of its components: while CTCs reflect tumor burden and correlate with micrometastases, ALRI provides additional information regarding hepatic injury and systemic inflammatory status. The integration of these parameters offers a multidimensional perspective on disease biology that neither biomarker can capture independently. Our study also confirmed tumor size and BCLC stage as independent predictors of RFS, consistent with their established roles in reflecting tumor burden and disease biology. Importantly, the CTC-ALRI score demonstrated superior sensitivity for predicting recurrence compared to these conventional prognostic factors, including tumor size and AFP level. Moreover, the CTC-ALRI score outperformed existing prognostic models such as BCLC stage, TNM stage, ERASL-pre, ERASL-post, SSCLIP scoring system and Korean models, further supporting its clinical utility. Furthermore, the CTC-ALRI staging system, utilizing only two parameters, is more concise and practical compared to traditional scoring systems. From a clinical perspective, the CTC-ALRI score enables risk-stratified surveillance and treatment strategies. Patients with a score of 0 may be managed with standard surveillance protocols, while those with a score of 1 warrant intensified monitoring with more frequent imaging and AFP surveillance. For patients with a score of 2, who demonstrated an 84.38% recurrence rate and median RFS of only 9.2 months, early adjuvant interventions such as TACE, targeted therapy, or immunotherapy should be prospectively evaluated to determine whether they can improve outcomes.

The biological basis underlying the association between the CTC-ALRI score and postoperative tumor recurrence is likely multifaceted; however, it must be emphasized that the present study was not designed to establish mechanistic causality, and the observed correlations should be interpreted as hypothesis-generating associations rather than direct mechanistic evidence. Elevated AFP expression—particularly at levels ≥400 ng/mL—has been independently associated with poor tumor differentiation and aggressive proliferative behavior in HCC (53, 54). The correlation between higher CTC-ALRI scores and elevated AFP levels observed in our cohort may therefore suggest that this composite score partly reflects the underlying biological aggressiveness of the tumor. Similarly, the progressive increase in Ki-67 expression across ascending CTC-ALRI score strata is consistent with the notion that higher scores may identify more proliferative tumor phenotypes with greater recurrence potential. Nonetheless, given that Ki-67 immunohistochemical data were available in only 46 patients (28.75% of the total cohort), these observations remain preliminary and necessitate confirmation in larger, adequately powered cohorts. Taken together, while these statistical associations are biologically plausible and internally consistent, they do not in themselves constitute mechanistic explanations for postoperative recurrence and should be regarded as the basis for future hypothesis-driven investigations. With respect to putative mechanistic pathways, it is speculated—on the basis of converging evidence from the existing literature rather than direct experimental data generated in the present study—that the CTC-ALRI score may be linked to postoperative recurrence partly through immune evasion mechanisms. CTCs have been implicated in multiple immunosuppressive processes: Sun et al. demonstrated through single-cell RNA sequencing that CCL5 overexpression in CTCs, regulated by p38-MAX signaling, recruits regulatory T cells (Tregs) to the tumor microenvironment, thereby facilitating immune escape and metastatic seeding (55). Additionally, platelet adhesion to CTCs enables cancer cells to evade natural killer cell-mediated killing through upregulation of the inhibitory immune checkpoint CD155, thereby promoting distant metastasis (56). Concurrently, elevated ALRI—reflecting both hepatocellular injury and lymphocyte depletion—may indicate an immunosuppressive systemic milieu characterized by impaired anti-tumor immune surveillance (57). Collectively, these observations support the hypothesis that higher CTC-ALRI scores may identify patients harboring a more immunosuppressive tumor-host interface, potentially predisposing to earlier postoperative recurrence. It must be underscored, however, that this mechanistic interpretation remains speculative, being extrapolated from independent lines of existing evidence rather than derived from direct experimental investigation within the present study. To rigorously elucidate the mechanistic relationship between the CTC-ALRI score and the tumor immune microenvironment, future studies should employ single-cell RNA sequencing to comprehensively characterize the transcriptional landscape of CTCs and tumor-infiltrating immune cell populations across CTC-ALRI score strata. Complementary multiplex immunofluorescence analyses would enable spatial mapping of immune cell composition and inhibitory checkpoint molecule expression within the tumor microenvironment. Such integrated approaches would provide the experimental foundation necessary to determine whether the CTC-ALRI score genuinely reflects immune evasion capacity, and to identify candidate immunotherapeutic targets for intervention in high-risk patients.

Several limitations of the present study warrant acknowledgment. First, the retrospective single-center design may introduce selection bias and limit the generalizability of the findings; furthermore, the use of internally optimized cutoff values may be subject to overfitting and optimism bias. Second, the relatively modest sample size precluded division into independent training and validation cohorts for external validation. Third, the use of single-time-point biomarker measurements, which may not fully capture dynamic biological changes. Fourth, the dichotomization of continuous variables, potentially leading to information loss. Fifth, the precise mechanistic basis underlying the association between the CTC-ALRI score and postoperative recurrence remains incompletely defined, and prospective studies incorporating molecular and immunological analyses are warranted to further characterize this relationship. Notwithstanding these limitations, to our knowledge, this is the first study examining the combined prognostic value of preoperative CTC and ALRI in HCC patients undergoing curative resection. These preliminary findings provide a foundation for future larger, multicenter, prospective studies with extended follow-up to validate our results and assess the clinical utility of this prognostic indicator. Additionally, further research should evaluate whether the CTC-ALRI score can guide risk-stratified treatment strategies and improve outcomes in high-risk patients.

## Conclusion

In conclusion, our study provides the first evidence that preoperative CTC-ALRI score serves as an independent prognostic factor for RFS in HCC patients undergoing curative resection. By integrating liquid biopsy (CTC enumeration) with systemic inflammatory status, this novel scoring system offers superior predictive performance compared to conventional prognostic factors. As a readily accessible and cost-effective biomarker, the CTC-ALRI score holds significant clinical potential for risk stratification, guiding personalized treatment strategies, and optimizing postoperative surveillance in HCC patients.

## Data Availability

The raw data supporting the conclusions of this article will be made available by the authors, without undue reservation.
