# Supplementary material for: A blood-biomarker based CTC-ALRI score predicts recurrence in hepatocellular carcinoma patients following curative resection
Source: Front Oncol. 2026 Mar 16;16:1788776. doi: 10.3389/fonc.2026.1788776 (PMC13033537; doi:10.3389/fonc.2026.1788776)
Supplement: Supplementary file 1 [file DataSheet1.docx]

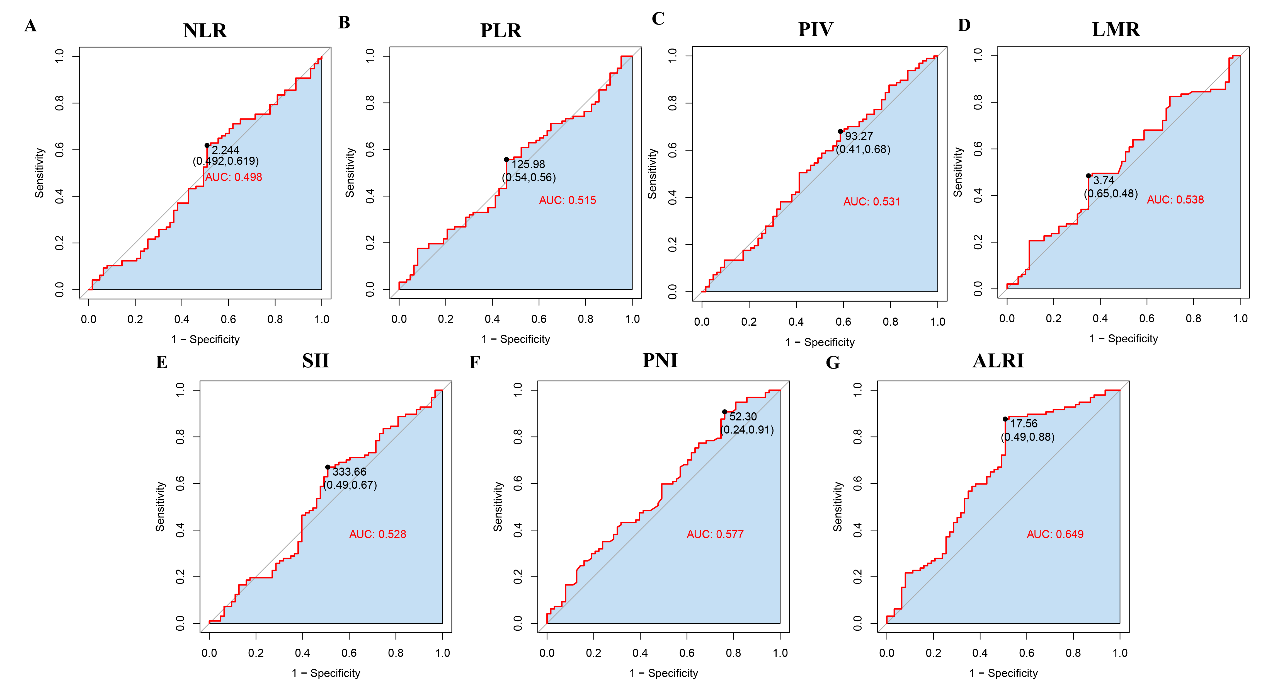


Fig.1 Determination of optimal cut-off value of the blood-biomarker. The ROC analysis of the optimal cut-off value of NLR (A), PLR(B), PIV(C), LMR(D), SII(E), PNI(F), and ALRI(G).
